# Leveraging agent-based modeling and a randomized intervention to advance childhood physical activity: A study protocol

**DOI:** 10.1371/journal.pone.0321301

**Published:** 2025-04-15

**Authors:** Matt Kasman, Adam B. Sedlak, Lydia Reader, William J. Heerman, Russell R. Pate, Amelie G. Ramirez, Evan C. Sommer, Shari L. Barkin, Ross A. Hammond

**Affiliations:** 1 Center on Social Dynamics and Policy, Brookings Institution, Washington, District of Columbia, United States of America; 2 Department of Mechanical and Aerospace Engineering, University of California San Diego Jacobs School of Engineering, San Diego, California, United States of America; 3 Division of Computational and Data Sciences, Washington University in St. Louis, St Louis, Missouri, United States of America; 4 Department of Pediatrics, Vanderbilt University Medical Center, Nashville, Tennessee, United States of America; 5 Arnold School of Public Health, University of South Carolina, Columbia, South Carolina, United States of America; 6 Institute for Health Promotion Research and Cancer Therapy and Research Center, University of Texas Health Science Center at San Antonio, San Antonio, Texas, United States of America; 7 Department of Pediatrics, Emory University School of Medicine, Atlanta, Georgia, United States of America; 8 Brown School at Washington University, St. Louis, Missouri, United States of America; 9 The Santa Fe Institute, Santa Fe, New Mexico, United States of America; PLOS: Public Library of Science, UNITED KINGDOM OF GREAT BRITAIN AND NORTHERN IRELAND

## Abstract

This study (1R01HD107002-01A1) protocol describes the planned creation and use of an agent-based model (ABM) of early childhood physical activity (PA). Successful early childhood PA interventions can potentially play an important role in both increasing overall population health as well as closing health disparities across subpopulations. At present, effective strategies for doing so are currently unknown. In large part, this is because PA determinants operate across levels dynamically, interact with one another, and can differ substantially across children. A complex systems approach—specifically, ABM—can be used to provide important insights about effect pathways driving child PA. Design of the proposed ABM will be based on high-quality extant research on childhood physical activity while allowing for the testing of hypotheses that extend beyond this body of literature. Its primary source of input data will be participants in GROW (NCT01316653), a completed cohort-based randomized controlled trial (RCT) that includes extensive longitudinal PA data collected from accelerometer observations of children from ages 3–9. We will iteratively test and improve upon an etiologic ABM of childhood PA, ensuring that it can satisfactorily reproduce micro- and macro-level influences and trends comparable to those seen in GROW. The tested ABM will then be used to extrapolate beyond the context of the GROW RCT, experimentally identifying potentially efficacious intervention strategies to improve childhood physical activity through program implementation or changes in policies and practices. We will use expert input to identify promising intervention approaches. We will use the model to systematically experiment with a wide array of different hypothetical combinations of intervention specifications and combinations. At the end of the model experimentation step, we expect to generate insights of broad applicability to the field of PA science regarding what might work, and for whom, in promoting PA and reducing disparities in these behaviors.

## Introduction

Almost half of U.S. adults have a preventable chronic disease, most of which could be improved with regular physical activity (PA) [1]. Numerous studies demonstrate a relationship between greater PA and favorable health outcomes for children and adults including metabolic, skeletal, psychosocial, cognitive, and cardiovascular health [2–10]. PA is a promising potential lever for addressing racial and ethnic health disparities due to both the high prevalence of negative health outcomes and low levels of PA within specific subpopulations; for example, on average, Hispanic women experience higher incidences of negative health outcomes and engage in less PA than other groups [11–17]. Significant health benefits accrue in adults who have been physically active throughout their lives [2–4,18,19]. Physical activity behaviors in childhood are associated with those same behaviors in adolescence and adulthood, meaning that even a moderate amount of PA in childhood can have immediate and long-term health benefits. Because PA behavior patterns are established at a young age, successful early childhood PA interventions can potentially play an important role in both increasing overall population health as well as closing health disparities across subpopulations [20–30].

Understanding the relative contributions of potentially modifiable determinants of childhood PA behaviors could lead to a better understanding of long-term health promotion strategies. While there are many programs and policies that seek to support sustainable patterns of health PA in early childhood, the most efficacious approaches are currently unknown, to the best of our knowledge. In large part, this is due to the vast number of factors that contribute to physical activity behaviors throughout childhood. Cross-sectional studies suggest a multilevel framework with influences at the macro-level (e.g., the built environment) [31–33], the meso-level (e.g., social environment) [34–37], and the micro-level (e.g., cognitive processes) [38–40]. That is, PA determinants operating across levels dynamically interact with one another, and can substantially shape the PA behaviors and development of children based on individual circumstances and experiences. These interrelated dynamics strongly suggest that childhood PA can be productively thought of as the result of a *complex adaptive system*. A complex adaptive system is one in which elements interact and change over time, generating system-level patterns that are often not linear, uniform, or intuitive [41]. Complex adaptive systems are characterized by the substantial presence of interdependence, adaptation, and heterogeneity [42]. In combination, these present serious challenges for traditional quantitative analytic techniques that rely on strict assumptions about the independence of observations [41–44].

Agent-based models (ABMs) have widely been used across fields of science to study complex adaptive systems, and members of our research team have successfully applied ABMs to study myriad public health topics [45–62]. ABMs are “bottom-up” computation simulation models, explicitly representing the actions of simulated “agents” (typically but not always individuals) over time, with system-level patterns emerging from an accumulation of micro-level behaviors [42–44]. These models are inherently dynamic and heterogeneous, allowing individuals with different attributes and behavioral traits to interact with one another and their environment, and to adapt their decision-making in response to interactions or changes in environment. While preliminary studies have demonstrated the potential application of ABM to PA behaviors in adults, only a few have examined PA in older children, and none have explored the interrelated mix of determinants that drive early childhood PA [63–69].

A key barrier to the development of ABMs of early childhood PA to date has been the need for sufficiently longitudinal, high-quality individual-level data. The Agent-based Computational Testing to Increase Vigorous Exercise (ACTIVE) project will develop the first data-driven, complex adaptive systems approach for modeling both PA behaviors in children (aged 3–9) and potential interventions to reduce PA disparities by leveraging the rich, high-quality data from the Growing Right Onto Wellness (GROW) RCT (NCT01316653) [70]. What follows is a description of the ACTIVE study protocol.

We will use ABM to pursue two primary research goals:

1) **Goal I: Develop an ABM that can reproduce observed patterns in early childhood PA.** This model will use high-quality data from a cohort-based randomized controlled trial (RCT) that includes extensive longitudinal PA data collected from accelerometer observations of children from ages 3–9. The focus of this research goal is on *inference*: iteratively testing and improving an etiologic ABM of childhood PA to reproduce micro- and macro-level influences and trends comparable to those seen in longitudinal cohort data.
2) **Goal II: Apply the ABM to counterfactual scenarios beyond those observed in the RCT.** The focus of this goal is *extrapolation*: beyond the settings in which the child PA data was collected during the RCT. These settings will shed light on what *could* happen to childhood PA in alternative exposure scenarios. In this way, the ABM will be a powerful “virtual laboratory” to gain insight into promising interventions policies and practices that could substantially, sustainably, and equitably improve childhood PA across diverse community contexts.

## Materials and methods

### Ethics statement

On 12/17/2024 the Emory IRB reviewed this study by expedited process. Due to the nature of this study, a complete waiver of HIPAA authorization and informed consent has been granted by the IRB.

### Approach overview

We will use extensive data collected during a completed RCT focusing on childhood obesity prevention and objective observation of childhood physical activity to develop and test an ABM of childhood physical activity. Classical statistical approaches typically make implicit assumptions (e.g., about independence between entities) that are incompatible with phenomena such as childhood PA. For example, children’s decisions to engage in PA are clearly intertwined as they observe one another, copy behaviors, and choose to play physically active games with one another. ABM is an appropriate methodological approach that can address some of these limitations to study childhood PA. ABM is a powerful analytical approach in which complex dynamic and spatial systems are studied “from the bottom up.” In an ABM, each individual actor in the system (i.e., each child) is explicitly represented as an autonomous computer software “agent.” This allows for substantial individual heterogeneity among agent attributes such as age, sex and environments such as parental physical activity and social settings experienced both inside and outside of the home. The agents are also given a set of hypothesized adaptive rules that govern their interactions with each other and with their environments (e.g., rules about how parental attitudes and activities affect children’s behaviors), allowing ABMs to effectively represent multiple simultaneous and potentially interdependent effect pathways. ABM is valued for the ability to capture: 1) feedback between multiple analytic levels; 2) direct and indirect effects on behaviors based on when and how individuals interact with each other 3) co-adaptation of individuals, groups, and the environments; 4) heterogeneity in both agent attributes and effect pathways; and 5) the possible impact of putative interventions via experimental manipulation of counterfactuals [43,44,71,72].

From the outset, our ABM will be designed to test hypotheses about heterogeneous effects, specifically whether, and in what ways, effect pathways differ throughout early childhood and across genders. Model relationships are anticipated to change over the course of childhood development, as will the influencing domains themselves (e.g., the built or social environments that children experience can change over time). The model will cover a time-period from early childhood (from age 3) to school-age (through age 8). The design of our model will be able to capture dynamic influences on PA, with some factors potentially becoming weaker or stronger during the course of early childhood development and socialization. We will also explore potential sources of heterogeneity in influence pathways across child characteristics. For example, the ways in which the built and home social environments combine may, on average, affect determinants of physical activity differently between girls and boys.

The nature of ABM is well-aligned with our goal of testing hypotheses about the etiology of early childhood PA. Because ABMs are computational, mechanistic models, the functional forms corresponding to every model element must be *fully specified.* Details of the model design such as which attributes are static or dynamic and how dynamic properties change over time need complete and logical and mathematical specifications [43,44]. Based on previous, successful ABM projects, we will use relevant empirical literature and guided collaborative discussions between content experts and modelers on our research team to translate research, experience, observation, and intuition into mathematical expressions [52,54,60,61,73].

### Model design

**Fig 1:**
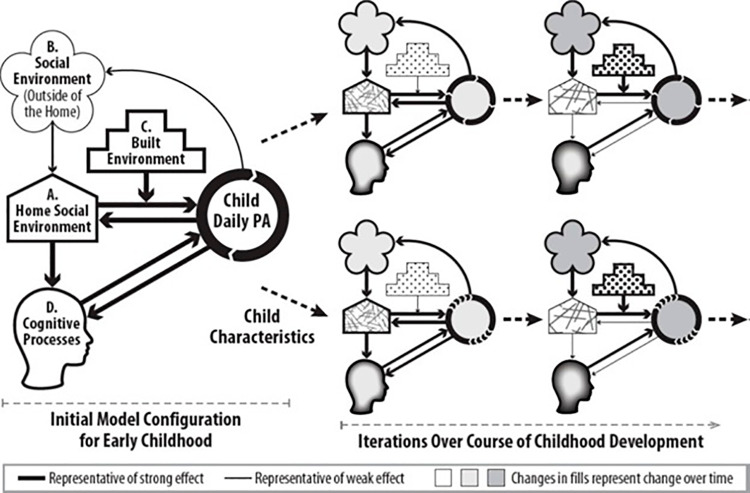
Conceptual framework: influences on children’s physical activity will initially be based on a review of extant literature. The interconnected systems of factors that drive physical activity patterns can change as children age and can differ substantially between children. Child-level influences change over time, with changes potentially interacting with child characteristics over the course of childhood development.

The design of our model will be based on high-quality extant research on childhood physical activity while allowing for the testing of hypotheses that extend beyond this body of literature. Our planned initial model design reflects the best currently available empirical evidence, theory, and expert guidance. The ABM will be designed to represent factors that research suggests have the strongest influence on childhood PA, as well as how these factors interact with one another, change over time, and can differ across children (Fig 1).

The model will simulate a cohort of young children with heterogeneous attributes interacting across heterogeneous social and physical environments. During the course of each simulation run, these simulated children can engage in varying degrees of physical activity. Levels of physical activity at any given point in time will be driven by:

A) The social environment in the home. This is primarily comprised of familial—especially parental—attitudes, and behaviors that can affect a child’s PA [34,74–77].
B) Social influences outside the home. Peers and adults outside of the family can be sources of (or barriers to) social support for PA [4,20,78–84].
C) The built environments that children experience. Defined as the human-made space in which people live, work, and play on a day-to-day basis. The built environment provides the environmental context in which physical activity occurs and can include factors affecting PA such as neighborhood walkability, safety, and access to green space [31–33,85–95].
D) Changes in cognitive processes that occur during child development. As children develop, their prefrontal cortex grows, enabling more sophisticated cognitive processes associated with decision-making, referred to as executive functioning. In particular our model will consider executive functioning which encapsulates self-regulatory cognitive processes related to engagement in PA including inhibitory control, attention, and reward sensitivity [39,40,96–100].

Our hypothesized model will incorporate two forms of feedback stemming from PA. The first is that physical activity can increase a child’s executive functioning; this contribution to development of cognitive processes increases a child’s capacity for engaging in future PA. The second is that engaging in physical activity in turn affects children’s social environments. For example, children enjoying PA could cause their peers to be more physically active or their parents to become more supportive of behaviors conducive to childhood PA. Our model will allow for effect pathways that display substantial heterogeneity across children. This is important because the makeup, strength, and characterization of effect pathways (alone or in combination) that influence PA can be expected to differ during childhood development and across characteristics such as age and gender [12,25,26,28,29,32,70,87,101–111]. Although informed by existing literature and preliminary studies, the process of operationalizing the ABM will by necessity entail testing many fine-grained hypotheses about the causal mechanisms that drive childhood PA that extend beyond existing literature.

### Data sources for model input

After initial model design, we will engage in model parameterization. During this process, we will assign values to each of the terms that appear in the model design. Data from the GROW RCT will be the primary source of input into parameter values. The GROW RTC was a childhood obesity prevention study that built on previous research with an emphasis on methodological rigor. It included multiple intervention components across disparate settings, had an unprecedented 3-year intervention window, and targeted children of underserved racial/ethnic groups and low socio-economic status [112]. GROW followed a cohort 610 children and parents or guardians from preschool to school-age while measuring a diverse set of phenotypes (i.e., anthropometrics, physiological, psychological, behavioral, socio-demographical, and environmental). Participants were enrolled between August 2012 and May 2014, with guardians providing informed, written consent for participation. Throughout the 3-year trial, attendance to all intervention sessions was high (>80%) and the overall retention rate was very high (>90%). Data on parent-child dyads were collected for 3 years on a rolling basis from 2012 to 2017 and included assessments of anthropometry, accelerometry, social network connections between parents, environmental conditions, and executive functioning. The study consisted of two-thirds preschool aged children who were high normal weight (BMI % ≥ 50% and <85%) and one-third who were overweight (≥85% and <95%). By study design, none were obese at the time of enrollment. Enrolled children and families were 90% Hispanic and 10% non-Hispanic Black. Children enrolled in the study from ages 3–6 years and were followed for 3 years (i.e., from ages 6–9 years). The GROW intervention provided 12 weekly skills-building group classes at community centers; followed by 9 months of phone call coaching and 24 months of cues to action to use the built environment for family health. The comparator condition provided school readiness and literacy promotion through a series of six workshops over 3 years.

GROW collected triaxial accelerometry at baseline and annually for 3 years from both children and parents using GT3X+ accelerometers with required minimum wear time of 360 minutes per day for at least four days including one weekend day. Sedentary, light, and moderate to vigorous physical activity (MVPA) data were defined using validated cut points for children and adults [28,113–115]. This allows us to examine physical activity objectively and in a granular way, distinguishing sedentary behavior from light, moderate, and vigorous physical activity. We also developed an algorithm to distinguish rest or sleep from sedentary behavior [116]. At baseline, GROW participants on average wore their accelerometers for 15 hours per day; wear time remained high throughout the study, providing unprecedentedly high-quality data on PA patterns.

Although we will rely primarily on GROW data for model input, these data are insufficient to fully characterize all of the model elements that we anticipate including in the final ABM design. In order for the model to represent the GROW cohort context as best as possible, we will supplement GROW data with other data sources hierarchically as follows:

1) Data from relevant external data sources. External data can be linked to GROW data either directly (for example, through geocoding) or indirectly by making implicit assumptions about the GROW cohort relative to samples in other datasets. External datasets will include the Competency-Based Approaches to Community Health (COACH) study, the National Survey of Children’s Health (NSCH), and the Child Opportunity Index (COI); NSCH and COI are both publicly available, and data from COACH will be restricted to aggregate descriptive statistics obtained upon request from study investigators [117–119]. We will also inform our model with publicly available data gleaned from Metro Nashville Public Schools, where many of the GROW participants attended school upon reaching school age.
2) Findings from relevant, high-quality literature. In some cases, we know or anticipate that previous studies—either observational or causal in nature—may provide parameter values. As with external data sources, this implicitly assumes compatibility of the samples studied and GROW.
3) Expert estimates. Members of our research team have extensive expertise in childhood PA that we will turn to parameterize the model as necessary and appropriate.
4) ABM calibration. The design of the model will incorporate elements for which specific estimates are unobserved and, in many cases unobservable (e.g., the mathematical form of functions that characterize children’s PA behaviors in specific settings). These parameter values will be obtained indirectly through calibration. That is, we will conduct extensive samplings of feasible parameter space and select parameter combinations that produce satisfactory matches between model outcomes of interest (e.g., child PA) and observed, real-world data.

A summary of our expected model input strategy is outlined in Table 1. Following best practices, we will share model input values derived from the above sources during dissemination of findings to ensure transparency and replicability.

**Table 1:** Summary of planned model input strategy.

Model Element	Expected Data Sources
**Agent Characteristics and Time Use**	
Agent Demographics (age, gender, family income)	GROW data
School Starting Age and School Year Schedule	Metro Nashville Public Schools - Entry Guidelines and Public Schools Calendar
Organized Physical Activity Participation	Competency-Based Approaches to Community Health (children <5) and National Survey of Children’s Health (children 5–9)
**Social Environments**	
Home Social Supportive Environment	Competency-Based Approaches to Community Health
Neighborhood Peers and Peer Influence	GROW data, expert input, calibration
School Peers and Peer Influence	GROW data, expert input, calibration
**Built Environments**	
Supportive and Prohibitive Home, Neighborhood, and School Physical Qualities and Features	GROW data, Child Opportunity Index data
**Cognitive processes**	
Initial Agent Executive Function Characteristics	GROW data, literature meta-analysis, expert input, calibration
**Model Dynamics**	
Combined Influence of Social Environments, Built Environments, and Cognitive Processes on Physical Activity (heterogeneous by age and gender)	Expert input, calibration
Influence of Child Physical Activity on Social Environments	Expert input, calibration
Change in Executive Function Characteristics (as a function of time and physical activity)	Literature meta-analysis, expert input, calibration

### Model development

After initial parameterization, the model will be instantiated in software architecture. Following established best practices from ABM and computer science, we will implement the ABM in Python, ensuring key functionality for computational modeling; extensibility with a wide array of pre-existing packages; and compatibility with standard statistical, GIS, and visualization software., including versioning and software testing [43,44,120–122]. In addition, conceptual testing via boundary adequacy tests, dimensional consistency, partial model testing, and extreme condition tests will be woven into the modeling process to ensure structural validity and internal consistency [123–126]. At the end of instantiation, we expect to have a well-specified, fully operationalized computational representation of the key pathways that drive physical activity in young children. This implementation will include structures to allow for heterogeneous impact of pathways across individuals and a data output structure that will be used to facilitate rigorous empirical testing.

### Model testing

Following model development, we will engage in rigorous and multifaceted model testing to assess its ability to produce expected output patterns. By comparing output from the completed model against empirically observed patterns of PA from GROW, we will be able to test the explanatory power of the model, and then explore how each component, independently and collectively, influences physical activity for different children across time and space within the context of GROW. These efforts will explicitly include the identification and incorporation of sources of substantial pathway heterogeneity. During this phase, we will explicate assumptions that underlie model design and subsequently subject these assumptions to sensitivity analyses to understand whether and to what extent they drive key findings. Following ABM best practices, the testing procedure will focus on assessing and improving upon the ability of the models to reproduce patterns and trends taken from GROW data [12,70,101–104,111]. Assessment of “fit” to GROW data will follow standard statistical tests appropriate to the type of data (e.g., time series, cross-sectional, associative). Specifically, data patterns that we will use to assess the model will include:

1) Distributions of PA minutes per day through early childhood. Due to the remarkably consistent extended wear time of accelerometers achieved in the GROW RCT, members of our research team have identified and published observations such as, on average at baseline, preschool-age children achieved more than 90 minutes of MVPA per day [104,111]. Consistent with prior literature, as children aged, their MVPA decreased from an average of 108 minutes (at baseline) to 84 minutes (1 year later), 82 minutes (2 years later), and 77 minutes (3 years later).
2) Overall gender disparities in PA. Another key finding that was facilitated by the notably long accelerometry wear-time of more than 15 hours per day during the GROW RCT was that boys typically obtained 14 minutes more of MVPA per day than girls. This seemingly small daily difference and the differential patterns that contribute to them could compound health effects as children age [70,104,111].

In addition to quantitative testing of the model, we will subject model design and behavior to qualitative assessment throughout the process to ensure consistent face validity. We will draw upon evidence from the GROW RCT and literature, alongside the extensive expertise of our research team and external Community Advisory Board to verify that the model meaningfully represents real-world fact patterns and dynamics as understood by researchers, intervention experts, practitioners, and community stakeholders, including parents of young children.

### Model experimentation

The ABM will be used as a “virtual laboratory” to extrapolate beyond the context of the GROW RCT, experimentally identifying potentially efficacious intervention strategies to improve childhood physical activity through program implementation or changes in policies and practices. We will apply the tested model to counterfactual settings and interventions beyond those observed during the GROW RCT so that insights can be generalized and provide guidance for future intervention efforts. A “counterfactual” setting refers to what would happen to a population’s health/behavior in alternative exposure scenarios [127,128]. ABMs provide a valuable opportunity to gain insight into potential policies and interventions through “in silico” experimentation (i.e., in the computer, via simulation) that goes well beyond any existing trial or dataset. We will have a valuable opportunity to gain insights that are relevant for the development of novel policies and interventions.

We will use expert input to identify promising intervention approaches. We will use the model to systematically experiment with a wide array of different hypothetical combinations of intervention effect magnitudes (i.e., “dose”), targeted recipients, and timing. In our previous work, we have found that policy interventions can have important synergies that make multiple small-dose intervention elements “supra-additive” in their impact—that is, effects that are greater than the sum of their effects individually [50,54,62]. We will explore the potential for such synergies here, exploring combinations of intervention approaches and leveraging the model’s ability to interact multiple pathways that drive PA across different childhood developmental stages. At the end of the model experimentation step, we expect to generate insights of broad applicability to the field of PA science regarding what might work, and for whom, in promoting PA and reducing disparities in these behaviors. These findings can suggest effective strategies for intervention selection and implementation.

### Study status and timeline

Iterative model design, development, and testing are underway. Experimentation is in the planning phase. Data collected during this study will be solely comprised of output from model runs during testing and experimentation. Data collection is expected to be complete by June 2025. Results (comprised of analyses of model output) will be available shortly thereafter, with dissemination activities described below expected to begin in July 2025.

## Discussion

### Limitations and expected challenges

The initial conceptual model of childhood PA was selected to parsimoniously reflect both key influences theorized and observed in extant literature as well as data collected during GROW. However, the components included are not exhaustive. If the model design described above does not achieve explanatory power (i.e., ability to satisfactorily reproduce patterns observed in GROW data), the assumptions made during design and parameterization will be systematically revisited and revised as we iteratively improve upon our model’s ability to reproduce expected patterns. We allow for the possibility that this might necessitate expansion of the model to include additional elements not described here or removal of elements that do not contribute to explanatory power. Members of our research team have encountered similar situations and have successfully navigated these challenges through a combination of substantial, ongoing literature review and model exploration, model testing, solicitation of stakeholder and expert input, and iterative adjustment [52].

The research described here entails combining data sources—including ones that represent expert estimates—and quantifying hypotheses about the specific characterization of dynamics that underlie childhood PA. All of this requires making many assumptions. Systematic exploration of model behavior will be undertaken as these assumptions are varied. The robustness of explanatory hypotheses from our etiologic exploration and of the findings from our experimentation with counterfactual scenarios will be quantitatively assessed (e.g., through the testing of model fit to data described above). This is a standard and essential best practice for all computational models, defending against over-specification and assumptions or parameters that may not be correct. This step will also provide additional important information to guide future data collection (by identifying the most important unknowns) and intervention development (by identifying targets that provide maximum impact on dynamics or are robust against contextual factors) [43,44,58,129,130].

### Dissemination

The nature of this research means that dissemination efforts should extend to multiple audiences. Findings that provide insight into the interrelated factors that underlie foundational early child PA are expected to be of interest to researchers and policy makers. The results of application of the model to counterfactual scenarios that shed light on policies and practices that can effectively increase child PA overall or reduce key disparities, and how successful intervention characteristics might differ across community contexts, should be made accessible to intervention experts, policymakers, and community stakeholders. Organizations that might be able to take effective action based on our findings include (but are not limited to) the Centers for Disease Control and Prevention; federal, state, or local departments of public health and education, the American Academy of Pediatrics, the National Institutes of Health, the Council of Governors, the United States Conference of Mayors, parks and recreation departments, and private or public community centers.

Finally, the nature of this research is highly innovative. To the best of our knowledge, the *ex post* extension of RCT data into the development and testing of an ABM is a novel approach to addressing research questions in public health. We believe that the use of a multidisciplinary research team including those involved with designing, fielding, and analyzing results from the RCT and those with extensive practice expertise in ABM can serve as a template for future synergistic combinations of RCT and complex adaptive systems research [131].
